# Seizures in children with neurofibromatosis type 1: is neurofibromatosis type 1 enough?

**DOI:** 10.1186/s13052-018-0477-x

**Published:** 2018-03-22

**Authors:** Claudia Santoro, Pia Bernardo, Antonietta Coppola, Umberto Pugliese, Mario Cirillo, Teresa Giugliano, Giulio Piluso, Giuseppe Cinalli, Salvatore Striano, Carmela Bravaccio, Silverio Perrotta

**Affiliations:** 1 Centro di Riferimento Pediatrico delle Neurofibromatosi, Dipartimento della Donna, del Bambino e di Chirurgia Generale e Specialistica, Università degli Studi della Campania “L. Vanvitelli”, Caserta, Italy; 2 Dipartimento di Scienze Mediche Traslazionali, Università “Federico II”, Napoli, Italy; 3 Centro Epilessia. Dipartimento di Neuroscienze, Scienze Riproduttive ed Odontostomatologiche, Università Federico II, Naples, Italy; 4 Dipartimento di Scienze Mediche, Chirurgiche, Neurologiche, Metaboliche e dell’invecchiamento, Università degli Studi della Campania “Luigi Vanvitelli”, Caserta, Italy; 5 Dipartimento di Medicina di Precisione, Università degli Studi della Campania “Luigi Vanvitelli”, Caserta, Italy; 6 Dipartimento di Neurochirurgia, Santobono-Pausilipon Children’s Hospital, Naples, Italy

**Keywords:** Seizures, Neurofibromatosis type 1, Microdeletion, Brain tumors, Unidentified bright objects, MMS

## Abstract

**Background:**

Neurofibromatosis type 1 (NF1) is related to a generally increased prevalence of seizures. The mechanism underlying the increased predisposition to seizures has not been fully elucidated. The aim of the study was to evaluate the role of NF1 in seizures pathogenesis in a cohort of children with NF1 and seizures.

**Methods:**

The medical records of 437 children (0–18 years old) with NF1 were reviewed. All children with at least one afebrile seizure were included. Demographic, clinical, neurological, *NF1* mutation status, and EEG data were collected along with brain magnetic resonance imaging. Depending on etiology, structural seizures have been identified and were further classified as NF1 related or not.

**Results:**

Nineteen patients (4.3%; 13 males) were included. NF1 was inherited in 7 (37.5%), with 3 maternal forms. Ten children with structural seizures were identified. Seven forms were identified someway related to NF1, two of which were associated to 17q11.2 microdeletion and hypoxic-ischemic encephalopathy. Any brain lesion that could explain seizures was found in nine patients, two third of these patients had a familiar history of epilepsy.

**Conclusions:**

Our results suggest seizures are more frequent in NF1 children (4.3%) than in general pediatric population (0.3–0.5%) and that are someway related to NF1 in half of patients. Facing seizures in NF1, the clinician should first exclude brain tumors but also other, and rarer NF1-related scenarios, such as hydrocephalous and vasculopathies. Children with non-structural seizures frequently had a family history of epilepsy, raising questions about the pathogenic role of NF1. They should be approached as for the general population.

## Background

Neurofibromatosis type 1 (NF1) is the most common neurocutaneous syndrome, [1] with a prevalence of about 1/3000 [2]. The classic manifestations of NF1 include café-au-lait macules (CALMs), axillary or inguinal freckling, iris Lisch nodules, neurofibroma, and bone dysplasia. NF1 patients are prone to developing brain tumors, especially optic pathway glioma (OPG). In addition, cerebrovascular diseases, such as Moyamoya syndrome (MMS), have been reported. Unidentified bright objects (UBOs) represent an intriguing NF1 lesion [3, 4]. UBOs are detected as focal lesions that show increased signal intensity on T2-weighted MRI scans, and they are more frequently found in children younger than 15 years old [5, 6]. Close to 90% of patients with NF1 have UBOs in their basal ganglia, thalamus, brainstem, and/or cerebellum [7].

NF1 is caused by mutations in the *NF1* gene, which is located at chromosome 17q11.2 [8].

Studies show that NF1 is related to a generally increased prevalence of seizures that ranges between 4% and 10% [9–11].

Recent data from patients with NF1 and seizures show that the increased prevalence of epilepsy in this population is mainly due to intracranial NF1-related tumors [11–13]. NF1 has also been associated with focal cortical dysplasia and hemimegalencephaly, both potentially related to epilepsy [13].

The potential pathogenic role of UBOs in seizures has been recently evaluated and excluded [10, 11, 14, 15]. Maternal inheritance of NF1 has been suggested as a predisposing factor for seizures [16, 17].

Mammalian target of rapamycin (mTOR) is actually retained a good candidate for epilepsy and epileptogenesis. In fact, recently animal studies results have suggested mTOR pathway plays a pathogenic role in epilepsy [18]. mTOR inhibitors reduce seizures in patients with tuberous sclerosis complex, a phacomatosis where a constitutively activation of mTOR exists [19]. Intriguingly, mTOR is constitutively activated also in NF1. Loss of neurofibromin leads hyperactivation of RAS, and subsequently of the RAF/MEK/ERK and phosphoinositide 3-kinase (PI3K)/mTOR pathways. Thereby NF1 brains might be per se hyperexcitable [20].

The aim of this study was to clarify the relationship of seizures and NF1 focusing on possible mechanisms of seizures. Towards this end, we retrospectively studied children 0–18 years old with NF1 who had seizures.

## Methods

We reviewed the clinical notes of patients diagnosed with NF1 according to the NIH criteria [21] who were followed at the Pediatric Tertiary Center of Neurofibromatosis in Naples, Italy, over a 25-year period (1990–2014). Patients were considered to have seizures if they experienced at least one afebrile seizure [22].

NF1 inheritance and genotype, age at diagnosis, and neurological phenotype were documented. Intellectual disability (ID) was defined as a measured IQ less than 70 (DSM IV) [23]. We recorded any family history of epilepsy, age of seizure onset, semiology and frequency of seizure according to the ILAE classification [24, 25], and we collected the patients’ electroencephalography (EEG) reports. Anti-epileptic treatment was recorded. If available, the seizure outcomes were registered, and the patients were classified as follows: group A, seizure-free for more than 1 year; group B, persistent seizures; and group C, loss to follow-up or unknown. A pediatric neurologist (PB) and two neurologists (AC; SS) with expertise in epilepsy reviewed the EEGs.

A neuroradiologist (MC) who was blinded to the clinical pictures of the patients reviewed the brain imaging studies. Brain tumors, vasculopathies, cortical dysplasia, and UBOs were documented. In patients with more than one exam, we considered results from brain imaging performed the closest to the time of seizure onset together with the most recent imaging results available for each patient.

Enrolled patients were considered to have a “structural seizure” if a brain structural lesion or injury was present (i.e. iatrogenic damage, tumors, mesial temporal sclerosis, moyamoya syndrome) with congruent electro-clinical pattern. Within this group, seizures were further divided in NF1 and non-NF1 related, considering whether the lesion was directly or indirectly associated to the genetic disease (i.e. brain tumors, surgery for NF1 intracranial complications). All the other patients with any recognizable brain lesion were classified as having “non-structural seizures”.

Three hundred and forty-five children and adolescent without epilepsy followed at our centre made the control group; 145 had undergone at least once brain imaging. Percentages were compared with chi-square or Fisher’s exact test. Odds ratio, and their two-side 95% confidence interval (CI), was estimated to identify risk or predictive factors.

## Results

A total of 437 clinical notes were reviewed. Of these, nineteen patients (4.3%; 13 males) had a personal history of seizures. The mean age at seizure onset was 5.5 years, and the mean age at diagnosis of NF1 was 6.2 years. Table 1 summarizes the patients’ demographic data, age at diagnosis of NF1, inheritance of NF1, family history of seizures, age at seizure onset, semiology of seizures, IDs, neuropsychiatric diseases, and neurological features.

**Table 1:** Clinical, radiological, and genetic characteristics of the pediatric NF1 patients included in the study

Patient ID/sex	Age at NF1 diagnosis (yrs)	Inheritance of NF1	NF1 mutation	Family history of seizures	Age at onset of seizures (yrs)	Seizures semiology	EEG features	Age at MRI (yrs)	NBOs presence and location	Other radiological findings	Treatment	Neuro cognitive profile and personal history	Out come	Structural vs non structural and NF1 vs non NF1 related epilepsy
1/M	4.9	P	c.2307_2308insC p.Thr770Hisfs*6	+ (P)	9.2	Oral automatisms, GTC	Focal, R posterior temporal region and secondary generalization	9.5	Absent	R mesial temporal sclerosis	CBZ, LEV		B	Structural non NF1
2/M	14.5	S	c.1185 + 1G > A p.Asn355_Lys395del	+ (Sister)	0.4	GTC, Ab	Normal	10	Absent	None	None	ID	C	Non structural
3/M	14.7	S	17q.11 microdeletion, Type1		0	ES	Focal, L, parieto -occipital	23	Absent	Symmetrical WMI adjacent to trigones	PH, PB, LEV	ID, neonatal hypoxia	A	Structural non NF1
4/M	13	S	c.5719G > T p.Glu1907*	+ (P)	13.8	GTC	Normal	NA			LEV	ID, SD	A	Non structural
5/M	7.7	S	NA		4.8	R clonic upper limb	Focal, left temporal	8.2	Bil Th	L basal ganglia gangliocytoma, OPG	Complete tumor excision, LEV, CL	ID	A	Structural NF1
6/F	0.5	S	NA		3.6	GTC	Normal	NA			VPA, CL	DD	A	Non structural
7/M	2.4	S	17q.11 microdeletion, Type1		9.7	GTC	Multifocal, asynchronous more prominent over the L hemisphere	10.1	Bil Th	None	VPA	ID, ASD, perinatal hypoxia	B	Non structural
8/M	8	P	c.6791_6792insA p.Tyr2264*	+ (M)	3.1	MyA; GTC	Generalized	9.5	Bil Th	None	VPA, PH, LEV	ID, SD	B	Non structural
9/F	16.7	M	Unknown; yet microdeletion, excluded		3.4	R upper limb rigidity	Multifocal posterior-temporal, L and R occipital	18.2	Bil Th; Bil Ce hemisphere; Br	L fronto-basal glioma	Radio therapy, CBZ	ID	C	Structural NF1
10/M	10	M	c.6085-2A > T p.Val2029Lysfs*7		11.9	My	Focal R parieto-occipital	11.9	Absent	Bilateral moyamoya	Bil indirect cerebral revascularization		A	Structural NF1
11/M	3.4	S	NA		5.2	GTC	Normal	5.2	Bil GP; Bil Th; L Ce hemisphere	Triventricular hydrocephalus, OPG	Ventriculo-peritoneal shunt		A	Structural NF1
12/F	5.2	M	c.7125delA p.Tyr2377Thrfs*20	+ (M)	4.1	Ab; GTC	Focal, R temporal	4.2	Bil Ce hemisphere	Symmetrical WMI adjacent to trigones, R corona radiata hyperintensity (Chemotherapy)	VPA	ID	B	Structural NF1
13/M	5	S	17q.11 microdeletion, Type3		0	Tonic motor activity and posturing, follone by cianosis	Focal, R temporal	4.1	Bil GP; Bil Th; Ce (L hemisphere and peduncle); Br	Symmetrical WMI adjacent to trigones	VPA, LEV	ID, neonatal hypoxia	B	Structural non NF1
14/M	1.7	S	c.4100_4103dupGTTT p.Tyr1369Phefs*6		6.5	GTC	Focal, R occipital	6.6	L GB; Bil Th; Ce (bil hemispheres and peduncles)	OPG	VPA	ID	C	Non structural
15/M	3	S	c.667 T > A p.Trp223Arg	+ (M)	5.6	GTC	Focal, R occipital	8	Bil GP; Ce (bilateral hemispheres and peduncles); Br	None	VPA		A	Non structural
16/M	5	P	c.5425C > T p.Arg1809Cys	+ (M)	3.2	My, GTC	Focal, L fronto-parietal	3.2	Absent	None	VPA		B	Non structural
17/M	0.6	P	c.3826C > T p.Arg1276*	+ (M)	2.4	Ab	Normal	3	Absent	Delayed myelinization	CBZ		B	Non structural
18/F	1.9	S	c.4381delA p.Ile1461*		7.3	Aphasia	Focal, L frontal	7.3	R GP; Bil Th; Ce (bil hemespheres); Br	L frontal cortico-subcortical jatrogenic encephalomalacia; OPG	Subtotal resection of the tumor, T, PB		B	Structural NF1
19/M	0.1	S	NA		10.6	Versive	Focal, L parieto-occipital	10.7	Absent	L rolandic pylocytic astrocytoma	Subtotal resection of the tumor, LEV	ID	A	Structural NF1

*Abbreviations*: *Ab* absent, *ASD* autism spectrum disorder, *Bil* bilateral, *Br* brainstem, *CBZ* carbamazepine, *Ce* cerebellar, *CL* clonazepam, *CPS* complex partial seizures, *DD* developmental delay, *ES* epileptic spasms, *F* focal, *GP* globus pallidus, *GTC* generalized tonic-clonic, *ID* intellectual disability, *L* left, *LEV* levetiracetam, *M* maternal, *My* myoclonic, *MyA* myoclonic-astatic, *NA* not available, *P* paternal, *PB* phenobarbital, *PH* phenobarbital, *PS* partial seizures, *R* right, *S* sporadic, *SD* speech disorder, *T* topiramate, *Th* thalamus, *VPA* sodium valproate, *WMI* white matter injuries, *A* Seizure-free for more than 1 year, *B* persistent seizures, *C* loss to or unknown follow-up

Neurofibromatosis was inherited from one affected parent in seven children (37%, three from an affected mother), while twelve patients (63%) had a sporadic form of NF1. Eight patients (42%) had family history of seizures. No statistically significant difference between the enrolled groups of epileptic patients and other children followed at our centre was found with respect to sex distribution or NF1 inheritance (respectively OR 1.75%, IC 95% (0.65–4.70) and OR 0.61%, IC 95% (0.24–1.59).

A molecular diagnosis of NF1 was available for 14 patients. Table 1 shows the type of NF1 mutation and the effects of mutation. Eleven patients had mutations in the *NF1* gene reported in Table 1. Any difference in terms of type of mutation was found respect of the control population. Three patients (patients 3, 7, and 13) had a 17q11.2 microdeletion that included the entire *NF1* gene; this finding will be discussed later.

The authors have already described three children: patient 10 [26] patient 11 [27] and patient 16 [28], respectively in paper focused on association of NF1 with MMS, hydrocephalus and a benign phenotype associated to Arg1809 substitution.

Neuroimaging examinations were available for all but two patients, patients 4 and 6. The patients’ neuroradiological features, age at MRI, and the presence and location of UBOs are reported in Table 1. More than one MRI were available for seven patients. Eleven patients had UBOs. In one patient, patient 1, the brain MRI control showed the disappearance of UBOs, with persistence of the seizures after anti-epileptic drug (AED) therapy. This patient also presented with mesial temporal sclerosis.

**Fig. 1:**
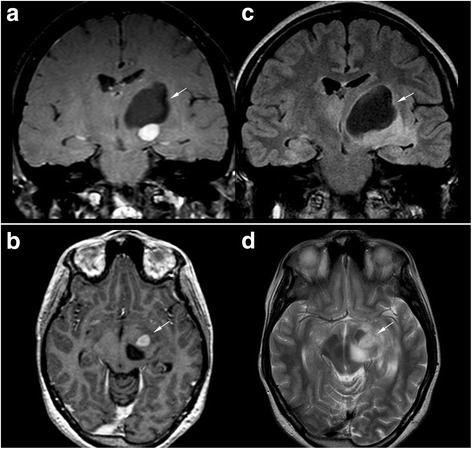
Magnetic resonance imaging of patient 5. (**a**, **b**) Gadolinium-enhanced T1-weighted images, (**c**) Coronal FLAIR image, and (**d**) axial FSE T2-weighted image of patient 5 show a large well-defined cystic mass in the left basal ganglia region (black arrow) with a eccentrically caudal solid mural nodule (white arrow). There is an associated mass effect on the 3rd and left lateral ventricles without hydrocephalus. Gadolinium-enhanced T1-weighted images show homogeneous marked enhancement (white arrow) of the solid portion in the subthalamic-mesencephalic region. Histological examination of the surgical specimen confirmed the diagnosis of gangliocytoma

**Fig. 2:**
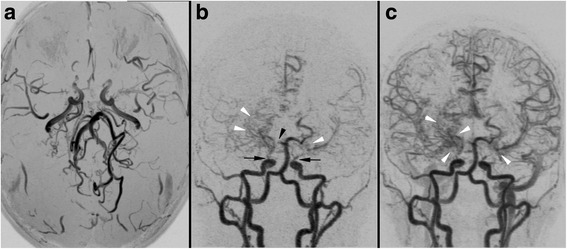
Magnetic resonance (MR) angiography 3D maximum intensity projection reconstructions in the patient 10. **a** Axial view of MR angiography of patient 10 without gadolinium. **b**, **c** Coronal views of dynamic contrast-enhanced MR angiography show bilateral terminal ICAs and right PCA stenosis/occlusion (arrow) with a fine vascular network in the basal ganglia, sylvian valley, and perimesencephalic cistern that compensates for the steno-occlusion (arrowhead indicates the moyamoya vessels). Suzuki staging criteria: III

**Fig. 3:**
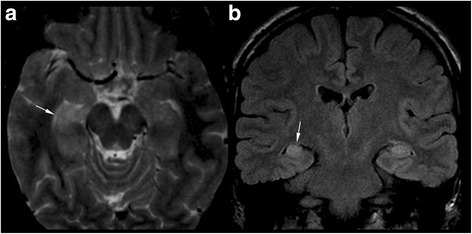
Magnetic resonance imaging of patient 1. **a** An axial FST T2-weighted image and (**b**) coronal FLAIR image of patient 1 shows slightly asymmetric hyperintense hippocampi (typical UBOs). The right hippocampus shows head volume loss (arrow) and flattened undulations (arrow in **a**) compared to the left thickened hippocampus. This is consistent with sclerosis

We identified 10 children with structural seizures and 9 with non-structural seizures. Most of patients with structural seizures were NF1 related (7/10). Two patients, patients 9 and 11, presented with just one seizure each; these seizures were due to frontobasal glioma and to hydrocephalus secondary to OPG, respectively. Patient 19 experienced focal seizures after surgery for a rolandic pylocitic astrocytoma. Patient 5 had focal seizures secondary to gangliocytoma (Fig. 1), and patient 10 somatomotor seizures secondary to Moyamoya syndrome (Fig. 2). In addition, one child, patient 12, had seizures and progressive cortical atrophy after a chemotherapeutic regimen to treat acute lymphoblastic leukemia. One patient, patient 18, had residual seizures associated with encephalomalacia due to surgical injury during debulking of an optic glioma involving the chiasmatic and hypothalamic region. One patient had mesial temporal sclerosis and focal seizures with secondary generalization (patient 1, Fig. 3).

Familiarity for seizures was analyzed for each of the two groups identified (Table 1). Family history of seizures was found in two patients (2/10, 20%) belonging to the structural epilepsy group. Indeed six patients with non-structural (6/9, 60%) had at least one relative affected too.

Patient 8 presented at 3 years of age with myoclonic-atonic and atonic seizures with generalized EEG patterns associated with severe impairment of cognitive functions, and a family history of seizures, figuring the well-defined electro-clinical syndrome Doose syndrome.

At least one EEG was available for all patients. Focal seizures with or without secondarily generalization were the most common type of seizures that were present in 11/19 children (58%). Patients with structural seizures had focal seizures in 8 cases; one patient had epileptic spasms (patient 3), one patient had tonic-clonic seizures (patient 11), with normal intercritical EEG, probably due to a secondary generalization.

Patients with non-structural seizures included 3 patients with focal EEG and clinical seizures semiology with probable secondary generalization. Three patients had generalized seizures and normal intercritical EEG data, and one patient with generalized seizures and generalized discharges in EEG. The other two were patient 7 and 8.

Sixteen patients received AED therapy: eight patients were seizure-free, three patients were lost to follow up, and patient 16 received therapy for less than 1 year at the time of this study (Table 1).

Five patients underwent surgery for intracranial NF1 complications (Table 1). Specifically, 3 underwent resection of brain tumors (patients 5, 18, 19). Bilateral indirect cerebral revascularization with multiple burr-holes was performed on patient 10; and patient 11 had a ventriculo-peritoneal shunt.

## Discussion

There is an intrinsic NF1-related risk of developing central nervous system (CNS) tumors, which partially explains the higher prevalence of seizures in patients with NF1 [12–14].

Here we described a group of children with NF1 and seizures in order to dissect the role of NF1 disease in the pathogenesis of seizures, with a focus on cases in which the seizures were apparently not strictly related to NF1. We also evaluated the NF1 inheritance pattern and genotype as well as the family history of seizures.

The prevalence of seizures in our cohort of children with NF1 was 4.3%, which is in line with previous reports [13, 17, 24]. There was a male predominance in our cohort (2.1:1) that was not significant when corrected by the male:female ratio of our general NF1 population (251 males/437 patients; *p* value ns). Ostendorf et al. [10] reported that individuals with NF1 and epilepsy were more likely to have inherited NF1 from their mothers.

There was no statistically significant difference in prevalence between patients with maternal and paternal inheritance or between patients with the familiar and sporadic forms of NF1 in our cohort (data not shown). Even though MRI data were scarce, no particular UBO localization was noted in our cohort. Moreover, not all patients who had MRIs showed UBOs at the onset of seizures. Along with more recent studies, this suggests that UBOs do not play any role in seizure pathogenesis [11, 12].

Focal findings were more commonly observed, in line with other studies [10, 13].

Within the structural seizures group of patients (10/19 patients) we report 7 patients with NF1-related causes of seizures including: brain tumors, MMS, hydrocephalus, cortico-subcortical atrophy, ischemic stroke and jatrogenic (surgical resection) (Table 1). Notably, both patients with MMS and hydroceplaus experienced resolution of seizures after surgery to address the underlying cause; they also did not need any AEDs. The 3 structural seizures forms not clearly related to NF1 included 2 patients with radiological signs of hypoxic–ischemic brain injury and epileptic encephalopathy as neonates. None of them had relatives with seizures, and both carried a 17q11.2 microdeletion. Brain MRI scans showed typical high-signal NF1 lesions hypoxic brain damage.

There was no statistically significant difference between the epileptic and non-epileptic patients who had undergone brain imaging (OR 1.64, IC 95%).

We also report the case of patient 1 who had typical temporal epilepsy; his father was similarly affected by NF1 and epilepsy. Temporal lobe T2-hyperintensities have been identified histologically as dysembryoplastic neuroepithelial tumors or gliosis [29]. Mesial temporal anomalies have been rarely reported in patients with NF1 [10, 30]. On the other hand, temporal epilepsy associated with mesial sclerosis is a common finding in the general epileptic population. Therefore the co-occurrence of mesial temporal sclerosis and NF1 is likely to be coincidental in our case.

We identified 9 children without a clear cause of seizures. Any recurring pattern of seizures was found. Even if all patients clinically experienced generalized seizures (especially Ab, GTC) we observed 3 focal and 1 multifocal EEG abnormalities (patients 15, 16, 17 and 7). This is in line with literature: focal abnormalities can occur without any recognizable injury at neuroimaging [10].

Among these children, we also report patient 8 who presented with a well-defined electroclinical syndrome, Doose syndrome. To our knowledge, this is the first NF1 patient with Doose syndrome to be reported in literature, thus this association seems to be coincidental.

In this group two third of children (6/9 patients) had a family history of seizures or epilepsy that segregated independently of NF1. Among the 3 patients with non-structural seizures and any familial history, patient 7 carried a 17q11.2 microdeletion.

Regarding the role of the *NF1* genetic background in seizures, we did not find any association between the type and location of the *NF1* mutations and seizures.

**Fig. 4:**
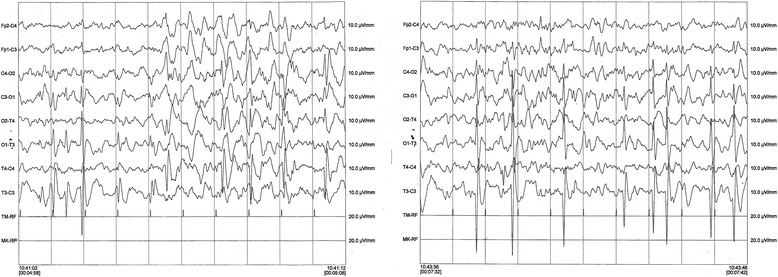
Awake EEG of patient 7. EEG of patient 7 shows multifocal asynchronous abnormalities characterized by high amplitude irregular sharp waves and polymorphic waves. These abnormali*ties* are more prominent over the left hemisphere

Yet we report three patients (patients 3, 7, and 13) carrying a 17q11.2 microdeletion that included the entire NF1 gene (Table 1). Patient 3 and 13 had hypoxic-ischemic encephalopathy (HIE), both of them had radiological evidence of hypoxia (Table 1). Patient 7 had a later onset of seizures at age of 9 years, with two tonic-clonic seizures and multifocal EEG activity (Fig. 4). He was born at term by a planned cesarean dissection because of maternal gestosis of late pregnancy. He was breastfed and early presented with severe psychomotor delay, ID and autism spectrum disorder. A brain MRI only showed UBOs. All these 3 patients had severe ID, which is in line with reports in the literature [31]. Even if the clinical picture differed in these three patients with microdeletion, surprisingly, in our study, the percentage of NF1 microdeleted patients (18%) was higher than that reported in the literature (5%) [32], and was also significantly higher than the percentage of microdeleted patients followed at our centre (3%) (OR 0.16) [33]. Van Minkelen et al. investigated the clinical and molecular characteristics of a large cohort of patients affected by NF1 and found that 3% of the patients had epileptic seizures but none had NF1 microdeletions. On the other hand, 9% of the patients with NF1 microdeletions, described by Venturin et al., experienced seizures [34]. These observations raise questions about whether the microdeleted patients are prone to develop seizures and if one of the genes localized to chromosome region 17q11.2 plays a key role in the development of seizures. The minimum deleted region shared by our three patients, which is found in both type 1 and type 3 microdeletions, does not include any genes that are known to be associated with epilepsy or seizures. One methylation-sensitive microRNA, MIR193A, which has been identified in temporal lobe epilepsy, is deleted in both type 1 and type 3 microdeletions [35]. It might be worthwhile to clarify whether MIR193A is involved in the pathogenesis of seizures, given that recent studies have shown that dysregulation of microRNA expression might be involved in epileptogenesis and brain excitability, and that pathophysiological features of temporal lobe epilepsy might be directly influenced by modulating individual microRNAs [36].

Patient 16 had an Arg1809 substitution that was described recently by our group as being related to a mild NF1 phenotype [26]. Arg1809 substitution is not known to be associated with seizures. The patient reported here has a family history of epilepsy that did not segregate with the NF1 trait.

Finally, we report an increased risk of ID in children with NF1 and seizures. Mild to severe ID was present in 11 patients (58%), with a higher prevalence than in the general NF1 population [17, 31]. This difference in prevalence rates may be partially due to the higher prevalence of microdeletion syndrome in our cohort or it could reflect a global phenomenon in epileptic children. In fact, independently by the underlying disorder, children with onset of epilepsy at an early age tend to present more severe cognitive impairment due [37].

Regarding drug control of seizures, some authors report that 60% of individuals with NF1 had good seizure control with just one AED or without AED therapy [14, 24]. Indeed, in our patients, seizures was globally well controlled by AEDs (Table 1) with none child with refractory epilepsy. Only the patient with Doose syndrome needed 3 drugs to control seizures, as frequently observed in this syndrome. We do no report cortical dysplasia or hemimegalencephaly in our cohort.

Out of brain lesions due to NF1, it is still unclear whether NF1 plays an intrinsic role in seizures in NF1 patients and why brains of individuals with NF1 are hyperexcitable and predispose to seizures [11, 13]. Some authors speculate a dysfunction of a variety of ion channels or a perturbation of GABAergic circuits may be involved in altering the inhibitory/excitation balance [11]. mTOR hyperactivation might be a further option [20]. Even if the hypothesis that NF1 itself might be the cause of a brain electrical dysfunction cannot be excluded, our observations suggest that other genetic susceptibility factors are likely to exist.

## Conclusion

In conclusion, when a child with NF1 presents seizures, the clinician should exclude brain tumors but also other, and rarer NF1-related scenarios, such as hydrocephalous and vasculopathy. In about half of cases, the seizures will not be clearly linked to NF1 and should be approached as for the general population.

Considering epilepsy without a recognizable cause alone it occurred with a prevalence of 2%, which is higher than prevalence in the general pediatric population (0.3–0.5%) [38]. Yet more then half of our patients had a family history of epilepsy.

Further study on patients with NF1 and non-structural seizures are desired to dissect if NF1 per se might predispose to epilepsy.

Although we found no relationship between the NF1 genotype or NF1 phenotype and seizures, yet here we report a significantly higher frequency of 17q11.2 microdeletion syndrome. Further studies are needed to investigate whether genes encoded in the microdeleted region are involved in the pathogenesis of seizures.

A multidisciplinary team approach is ideal to children with NF1 and seizures, so that the child can be referred to appropriate specialists for a diagnostic work-up or for treatment or genetic counseling.

## Abbreviations

AED: Anti-epileptic drugs
ASD: Autism spectrum disorder
CALMs: Cafè-au-lait macules
CNS: Central nervous system
EEG: Electroencephalography
HIE: Hypoxic-ischemic encephalopathy
ID: Intellectual disability
IQ: Intelligence quotient
IS: Infantile spasms
MRI: Magnetic resonance imaging
NF1: Neurofibromatosis type I
NIH: National Institutes of Health
OPG: Optic pathway glioma
TSC: Tuberous sclerosis complex
UBO: Unidentified bright objects
